# Degradation of topological surface state by nonmagnetic S doping in Sr_*x*_Bi_2_Se_3_

**DOI:** 10.1038/srep45565

**Published:** 2017-03-30

**Authors:** Hui Huang, Juanjuan Gu, Min Tan, Qinglong Wang, Ping Ji, Xueyou Hu

**Affiliations:** 1 Department of Electronic Information and Electrical Engineering, Hefei University, Jinxiu Road 158, Hefei 230601, Peoples Republic of China

## Abstract

Research on possible topological superconductivity has grown rapidly over the past several years, from fundamental studies to the development of next generation technologies. Recently, it has been reported that the Sr_*x*_Bi_2_Se_3_ exhibits superconductivity with topological surface state, making this compound a promising candidate for investigating possible topological superconductivity. However, whether or not the topological surface state is robust against impurities is not clear in this system. Here we report a detailed investigation on the lattice structure, electronic and magnetic properties, as well as the topological superconducting properties of Sr_*x*_Bi_2_Se_3−*y*_S_*y*_ samples. It is found that the superconducting transition temperature keeps nearly unchanged in all samples, despite of a gradual decrease of the superconducting shielding volume fraction with increasing S doping content. Meanwhile, the Shubnikov-de Hass oscillation results of the Sr_*x*_Bi_2_Se_3−*y*_S_*y*_ samples reveal that the topological surface states are destroyed in S doped samples, suggesting the topological character is degraded by nonmagnetic dopants.

The topological quantum matter states have become one of the hottest research topics in condensed matter physics and materials science communities^1^,^2^,^3^,^4^,^5^,^6^,^7^,^8^,^9^,^10^,^11^,^12^,^13^. The topological insulating state, the Dirac semimetal state, as well as the Weyl semimetal state have been theoretically proposed and experimentally proven in a variety of bulk materials in recent years^4^,^7^,^8^,^9^,^10^. Besides the above mentioned topological quantum states, it has been proposed that a novel topological superconducting state may emerge at the boundary between a superconductor and a topological insulator^3^. The topological superconducting state is featured with a full pairing gap in the bulk and gapless surface states at the surfaces. The topological superconductor is believed to be an ideal platform for searching of Majorana Fermion, a long-sought yet elusive quasiparticle which has been extensively investigated in high-energy physics for many years.

The searching of topological superconducting state in a real material has been proven to be a big challenge. In the past decade, there are tremendous efforts aiming to realize the topological superconcuting state^14^,^15^,^16^,^17^. In particular, the discovery of superconductivity in Cu-intercalated Bi_2_Se_3_ topological insulator has attracted much attention, because large-size Cu_*x*_Bi_2_Se_3_ superconducting single crystals can be grown. A lot of theoretical study and experimental work have been performed on this compound in order to realize possible topological superconductivity in bulk samples^18^,^19^,^20^,^21^,^22^. However, whether or not the Cu_*x*_Bi_2_Se_3_ is a topological superconductor is still controversial. For example, the point-contact spectroscopy measurements have clearly shown the presence of zero-bias conductance peaks from the Majorana bound states at the surface edges^19^. On the contrary, the scanning tunneling spectroscopy measurements reveal a fully-gapped feature in the density of states and there is no in-gap state, possibly suggesting that the superconducting state in the Cu_*x*_Bi_2_Se_3_ samples is topologically trivial^21^. Thus it is of particular importance to investigate the properties of possible topological superconducting state in alternative compounds. Recently, it has been reported that by intercalation of alkaline earth element Sr into the Bi_2_Se_3_ topological insulator, superconductivity with large superconducting volume fraction can be realized in Sr_*x*_Bi_2_Se_3_ system^23^. It has also been experimentally proven that the Dirac point and the topological surface states are well-preserved in the Sr_*x*_Bi_2_Se_3_ samples^23^,^24^,^25^,^26^. Furthermore, angle-dependent resistivity measurements on Sr_*x*_Bi_2_Se_3_ single crystals by different groups have revealed apparent two-fold anisotropy, indicating rotational symmetry breaking in this compound^27^,^28^. The nodeless and two-fold symmetric superconducting gap is consistent with the prediction of topologically nontrivial superconductivity in Sr_*x*_Bi_2_Se_3_. These facts suggest that the Sr_*x*_Bi_2_Se_3_ compound could serve as an important material platform for the investigation of topological superconductivity.

In this work, we perform a systematic investigation on the crystal lattice, the transport behavior, as well as the topological superconducting properties of a series of Sr_*x*_Bi_2_Se_3−*y*_S_*y*_ single crystal samples. It is found that the isovalent S doping at the Se site does not lead to any noticeable change in the charge carrier density, which is of particular importance in identification of the intrinsic effects of S doping in a topological compound. The nonmagnetic S doping results in a gradual decrease of the superconducting shielding volume fraction of the Sr_*x*_Bi_2_Se_3−*y*_S_*y*_ compound, while the onset of the superconducting transition temperature keeps nearly unchanged in all samples. Furthermore, the analysis of the Shubnikov-de Hass oscillation data reveals that the nonmagnetic S-doping can also destroy the topological surface states of the samples. These results demonstrate that the topological feature of the Sr_*x*_Bi_2_Se_3_ system is sensitive to nonmagnetic impurities.

In order to know to what extend the nonmagnetic S ions are incorporated into the Sr_*x*_Bi_2_Se_3_ lattice, we perform energy dispersive x-ray spectrometry analysis on the S-doped Sr_*x*_Bi_2_Se_3_ samples. The comparison between nominal and real compositions of the Sr_*x*_Bi_2_Se_3−*y*_S_*y*_ samples is listed in Table 1. It can be seen from Table 1 that the actual Sr contents in all samples are quite close to 0.066, consistent with previous reports^23^. It is also clear that the actual S doping content in each sample is very close to the nominal doping content, meaning that the nonmagnetic S ions can easily substitute the Se ions.

Figure 1 shows the powder x-ray diffraction patterns of the Sr_*x*_Bi_2_Se_3−*y*_S_*y*_ samples as well as a representative single crystal x-ray diffraction pattern of the *y* = 0.2 sample. From the single crystal XRD pattern it can be seen that only the (00*l*) diffraction peaks appear, suggesting that the crystallographic *c*-axis is perpendicular to the shining surface. For all the diffraction peaks, the full width at half maximum (FWHM) is less than 0.06°, indicating the high-quality of the samples. From the powder XRD patterns we notice that all the diffraction peaks can be well-indexed in rhombohedral *R*-3*m* space group with no unidentified peaks. The lattice parameters for the parent compound are *a* = 4.1428 Å and *c* = 28.563 Å, which are similar to previous reported values^23^. For all the peaks, they exhibits very slight shift to higher angle with increasing S doping content, meaning that both the *a*-axis and the *c*-axis lattice constants are shrunk upon S doping. The variation of lattice parameters with increasing S doping is given in Table 1. The decrease of both *a* and *c* lattice parameters is consistent with the fact that the radius of S^2−^ (1.02 Å) is smaller than that of Se^2−^ (1.16 Å). The monotonous decrease of the lattice parameters with increasing S doping suggests that the S ions are substantially incorporated into the Sr_*x*_Bi_2_Se_3_ crystal lattice.

The temperature dependence of in-plane resistivity of the Sr_*x*_Bi_2_Se_3−*y*_S_*y*_ samples is given in Fig. 2. For the samples with S doping level *y* ≤ 0.3, they exhibits metallic-like behavior at the normal state. The normal state resistivity gradually increases with increasing S doping content, meaning that the isovalent S dopants introduce some random disorder which can scatter the motion of the charge carriers. The inset of Fig. 2 shows an enlarged view of the resistivity near the supercoducting transition temperature. It can be seen that the onset temperature of the superconducting transition (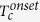) is about 2.9 K for the undoped Sr_0.066_Bi_2_Se_3_ sample, which is consistent with previous reports^23^,^24^,^25^,^26^,^27^,^28^. The width of the superconducting transition is less than 0.3 K, suggesting the high-quality of the single crystal sample. With the introducing of S dopants, it is found that the superconducting transition becomes weakened. For the samples with *y* ≥ 0.2, the resistivity does not reach zero even when the temperature is down to 1.8 K. Despite of the fact of the gradual depression of superconductivity with increasing S dopants, it is interesting to notice that the 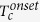 values of the S doped samples are all close to 2.9 K. In other words, the 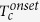 value keeps nearly unchanged with increasing S doping.

In order to know whether or not the S doping leads to any change in the charge carrier concentration of the Sr_*x*_Bi_2_Se_3_ compound, we determine the charge carrier density of the Sr_*x*_Bi_2_Se_3_ parent sample and the S-doped samples which is derived from the Hall coefficient measurements. The variation of charge carrier concentration (*n*_*e*_) as the function of temperature for the *y* = 0, 0.2, and 0.4 samples is given in the inset of Fig. 2. For the undoped Sr_0.066_Bi_2_Se_3_ sample, the *n*_*e*_ value is 2.14 × 10^19^ cm^−3^ at room temperature, which is consistent with previous reports^23^,^24^,^25^,^26^. We notice that the introduction of S in the Sr_*x*_Bi_2_Se_3−*y*_S_*y*_ compound does not lead to any significant change in the charge carrier concentration. For example, the *n*_*e*_ value in the *y* = 0.4 sample is 2.03 × 10^19^ cm^−3^ at room temperature, which is comparable with that of the undoped sample. Thus it can be concluded that the suppression of superconductivity by S doping is not originated from the change in charge carrier concentration.

In order to see clearly how the nonmagnetic S doping suppresses the superconductivity of the Sr_*x*_Bi_2_Se_3_ system, we perform the measurements of the temperature dependence of magnetic susceptibility (*M* ~ *T*) of the Sr_*x*_Bi_2_Se_3−*y*_S_*y*_ samples. The results are shown in Fig. 3. The onset superconducting transition temperature determined from the *M* ~ *T* curve of the *y* = 0 sample is about 2.85 K. And the shielding volume fraction increases sharply with decreasing temperature, indicating a very good diamagnetic behavior. It can be seen that the shielding superconducting volume fraction of the undoped Sr_0.066_Bi_2_Se_3_ sample is about 90.3% at 1.8 K, which is consistent with previous reports^23^,^24^,^25^. With increasing S doping, the shielding volume fraction gradually decreases, suggesting the suppression of superconductivity. For the *y* = 0.4 sample, the shielding volume fraction is zero, meaning a completely depression of superconductivity. It is worth noticing that despite of the gradual decrease of shielding fraction, the onset superconducting transition temperature determined from the *M* ~ *T* curve keeps nearly unchanged at about 2.85 K for all samples. This fact suggests that the S dopants destroys the superconductivity of the Sr_*x*_Bi_2_Se_3_ system locally. In other words, the superconductivity is completely destroyed in a small area near the S dopants, while the areas far away from the S dopants remain intact. The locally depression of superconductivity has also been discovered in some doped cuprate and iron-based superconductors^29^,^30^. This locally destroyed superconductivity probably means an unconventional superconductivity.

In order to know whether or not the nonmagnetic S dopants destroy the topological surface state of the Sr_*x*_Bi_2_Se_3_ system, we perform the Shubnikov-de Hass (SdH) oscillation measurements on both the undoped and the S-doped samples. The analysis of the quantum oscillation data under magnetic field has recently been widely employed in the investigating of topological materials^31^,^32^,^33^,^34^. Figure 4(a–c) show the magnetic field dependence of resistivity of the *y* = 0, *y* = 0.2, and *y* = 0.4 samples, respectively. The temperature is kept at 2 K. For the undoped Sr_0.066_Bi_2_Se_3_ sample, it can be seen that the superconductivity is rapidly killed with increasing external magnetic field. When the applied magnetic field is larger than 0.36 T, the transition from the superconducting state into normal state is finished. The Sr_0.066_Bi_2_Se_3_ sample exhibits positive magnetoresistance. A profound oscillation appears when the magnetic field is higher than 7 T, suggesting the high-quality of the single crystal sample and the high mobility of the charge carriers. We analyze the oscillation signal by subtracting the background and plot the oscillation data in Fig. 4(d). It can be seen that the oscillation is periodic against 1/*B*. The simple pattern shown in Fig. 4(d) gives a single frequency of F = 142.5 T. For the S doped samples, as can be seen from Fig. 4(b,c), a clear SdH oscillation signal appears when the applied magnetic field is higher than 8 T. The oscillation frequencies in the *y* = 0.2 and *y* = 0.4 samples are F = 150.2 T and F = 148.1 T, respectively. It can be seen that the introduction of S hardly affects the oscillation frequency, meaning that the S dopants does not alter the Fermi surface topology of the Sr_*x*_Bi_2_Se_3_ compound.

In a solid state material, any closed cyclotron orbit is quantized under an external magnetic field *B*, according to the Lifshitz-Onsager quantization rule


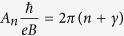


where *A*_*n*_ is the extremal cross-sectional area of the Fermi surface (FS) related to the Landau level (LL) *n*. And *γ* represents an additional Berry’s phase. The additional Berry’s phase (*γ*) in a non-topological material is zero. For an ideal topological quantum material with surface states, the additional Berry’s phase *γ* should be close to 1/2. We analyzed the SdH oscillations of the Sr_*x*_Bi_2_Se_3−*y*_S_*y*_ samples by plotting the Landau index versus the inverse of the magnetic field (1/B). The results are given in Fig. 5. For the undoped Sr_0.066_Bi_2_Se_3_, all the data fall into a straight line and the liner extrapolation gives an intercept at *γ* = 0.53 (Fig. 5(a)). The existence of a nontrivial Berrys phase (*γ* = 0.53) suggests the existence of surface states in the Sr_*x*_Bi_2_Se_3_S system, which is consistent with previous SdH oscillation and angle-resolved photoemission spectroscopy results^23^,^24^,^25^. For the *y* = 0.2 sample, the obtained *γ* value is 0.21, which is neither close to 1/2 nor close to 0. Thus it is difficult to claim whether or not there are surface states in the *y* = 0.2 sample. As can be seen from Fig. 5(c), the obtained *γ* value is zero in the *y* = 0.4 sample, meaning the completely disappearance of surface states in this sample. These results suggest that the topological features in the Sr_*x*_Bi_2_Se_3_ compound is gradually destroyed with nonmagnetic S doping. Thus the present study reveals that the topological character can be sensitive to nonmagnetic S dopants in Sr_*x*_Bi_2_Se_3_ compound.

A recent study reveals that the incorporation of S in the middle layer of the quintuple-layer crystal lattice of Bi_1.08_Sn_0.02_Sb_0.9_Te_2_S decreases the absolute energy of the valence band and makes the Dirac point isolated in energy from the bulk states^35^. In Bi_1.08_Sn_0.02_Sb_0.9_Te_2_S system, the topological surface state is robust against S incorporation. The fact of the degradation of surface state in S-doped Sr_*x*_Bi_2_Se_3_ compound is interesting and needs further investigation. A systematic angle resolved photoemission spectroscopy study would probably reveal the physical reason.

In conclusion, we perform a systematical investigation on the superconductivity and topological surface states of the Sr_*x*_Bi_2_Se_3_ compound with nonmagnetic S doping. The superconducting volume fraction is gradually decreased with increasing S doping concentration while the onset superconducting transition temperature keeps nearly unchanged, suggesting that the nonmagnetic S dopants destroy the superconductivity locally. Interestingly, we find that the nonmagnetic S dopants destroys the topological surface states of the Sr_*x*_Bi_2_Se_3_ system.

## Methods

Single crystal of a series of S-doped Sr_*x*_Bi_2_Se_3_ were prepared using self-flux method as reported previously^23^. Stoichiometric mixtures of Bi powder, Sr piece, Se powder and S powder were sealed in evacuated quartz tubes. In order to achieve a reliable conclusion, we keep the nominal Sr content at *x* = 0.16 in all S-doped samples. The tubes were heated at 850 °C for 48 h, followed by a slow cooling to 600 °C at a rate of 2.5 °C/h. After that, the furnace was shut down and the samples were cooled down with furnace. The chemical compositions of the obtained crystals were examined using energy dispersive x-ray spectrometry (EDX) analysis, which was performed using Oxford SWIFT3000 spectroscopy equipped with a Si detector. For each sample, about twenty different points were randomly selected in the EDX measurements and the average was defined as the real composition. The obtained crystals were characterized by powder x-ray diffraction (XRD) and x-ray single crystal diffraction with Cu K_*α*_ radiation at room temperature. The temperature dependence of resistivity and Hall coefficient were measured in a commercial Quantum Design physical property measurement system (PPMS-14 T) system. In order to get reliable results, we mount the *y* = 0, *y* = 0.2, and *y* = 0.4 samples in one sample holder in the Shubnikov-de Hass oscillation experiments. The [001] crystal axis have been carefully aligned to ensure that angle between the applied magnetic field and the [001] crystal axis is identical for the three samples during the experiments. Magnetic properties were performed using a superconducting quantum interference device magnetometer (SQUID). The applied magnetic for both zero-field cooling process and field-cooling process is 2 Oe.

## Additional Information

**How to cite this article**: Huang, H. *et al*. Degradation of topological surface state by nonmagnetic S doping in Sr*_x_*Bi_2_Se_3_. *Sci. Rep.* **7**, 45565; doi: 10.1038/srep45565 (2017).

**Publisher's note:** Springer Nature remains neutral with regard to jurisdictional claims in published maps and institutional affiliations.

## Figures and Tables

**Figure 1:**
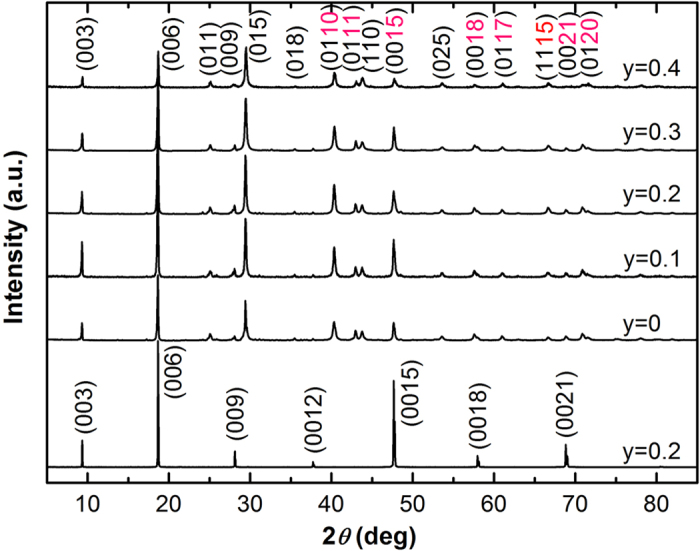
Powder x-ray diffraction patterns of the Sr_*x*_Bi_2_Se_3−*y*_S_*y*_ samples. The bottom one is a representative single crystal x-ray diffraction pattern of the *y* = 0.2 sample.

**Figure 2:**
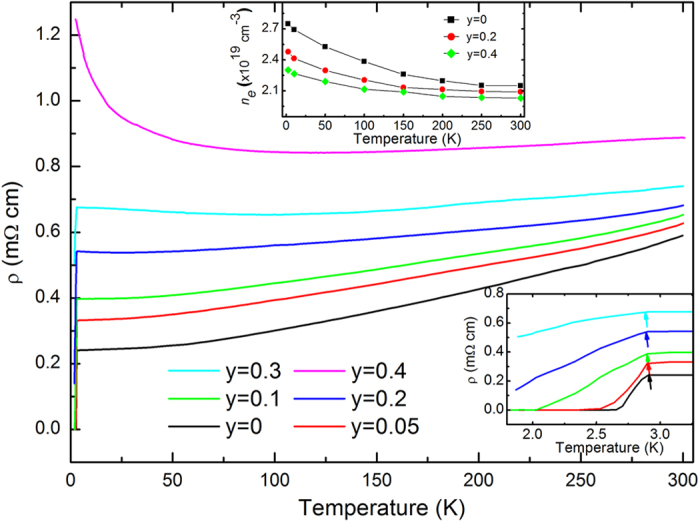
Temperature dependence of in-plane resistance of the Sr_*x*_Bi_2_Se_3−*y*_S_*y*_ samples. The lower inset shows an enlarged view near the superconducting transition region. The upper inset gives the variation of charge carrier concentration as the function of temperature and S doping.

**Figure 3:**
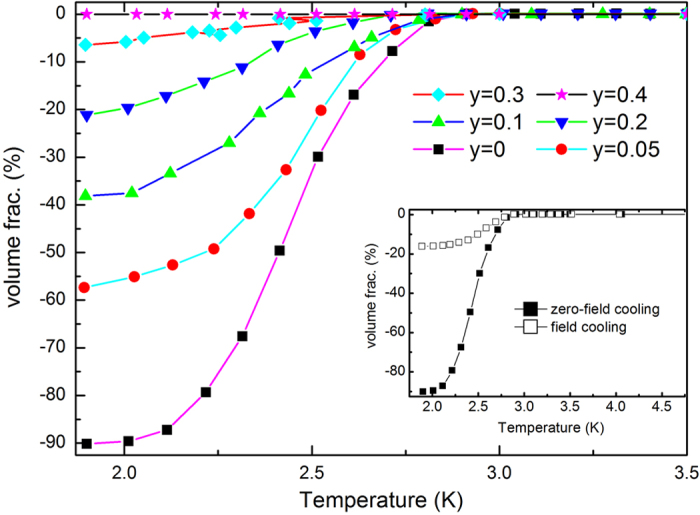
The temperature dependence of magnetic susceptibility of the Sr_*x*_Bi_2_Se_3−*y*_S_*y*_ samples measured under zero-field cooling process. The applied magnetic field is 2 Oe. The inset gives a comparison between the zero-field cooling process and field-cooling process of the undoped Sr_0.066_Bi_2_Se_3_ sample.

**Figure 4:**
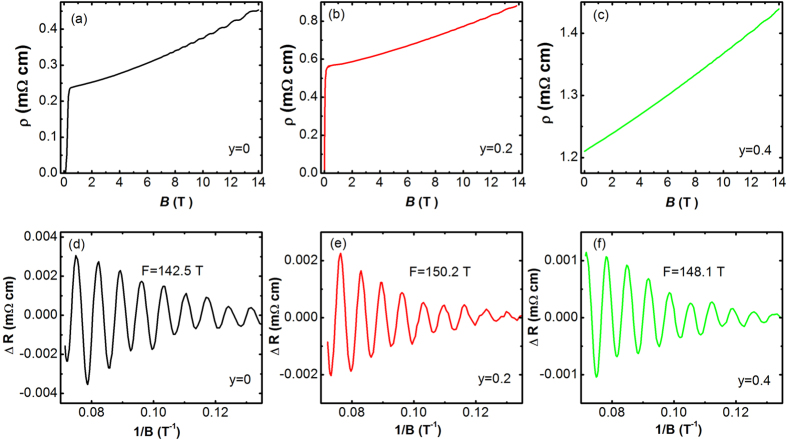
(**a**–**c**) The magnetic field dependence of resistivity of the Sr_*x*_Bi_2_Se_3−*y*_S_*y*_ samples with (**a**) *y* = 0, (**b**) *y* = 0.2, and (**c**) *y* = 0.4. The temperature is kept constant at 2 K. (**d**–**f**) The Shubnikov-de Hass oscillation patterns of the (**d**) *y* = 0, (**e**) *y* = 0.2, and (**f**) *y* = 0.4 samples.

**Figure 5:**
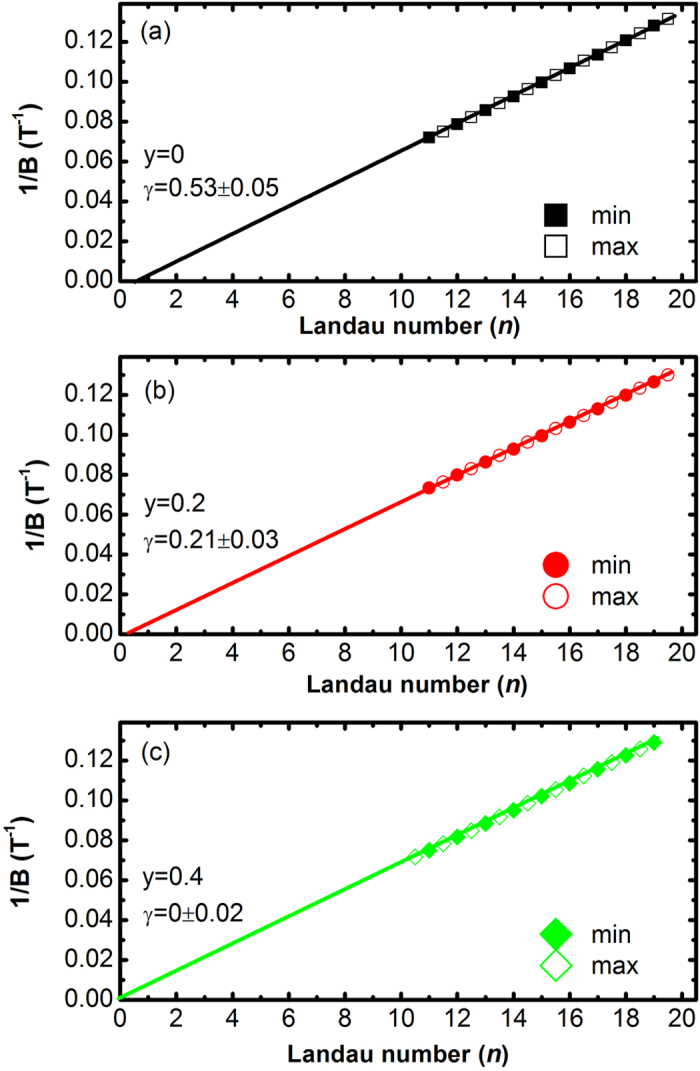
The Landau number (*n*) plotted against 1/*B* for the (**a**) *y* = 0, (**b**) *y* = 0.2, and (**c**) *y* = 0.4 samples. The closed symbols denote the integer Landau number (the minimum of ΔR), and the open symbols indicate the half integer index (the maximum of ΔR).

**Table 1:** The comparison between nominal and real compositions of the Sr_*x*_Bi_2_Se_3−*y*_S_*y*_ samples as well as the lattice parameters of the samples.

Nominal composition	Real composition	*a* (Å)	*c* (Å)
Sr_0.16_Bi_2_Se_3_	Sr_0.066_Bi_2_Se_3_	4.1428	28.563
Sr_0.16_Bi_2_Se_2.95_S_0.05_	Sr_0.066_Bi_2_Se_2.95_S_0.05_	4.1418	28.56
Sr_0.16_Bi_2_Se_2.9_S_0.1_	Sr_0.065_Bi_2_Se_2.9_S_0.1_	4.1409	28.555
Sr_0.16_Bi_2_Se_2.8_S_0.2_	Sr_0.066_Bi_2_Se_2.8_S_0.2_	4.1381	28.541
Sr_0.16_Bi_2_Se_2.7_S_0.3_	Sr_0.066_Bi_2_Se_2.71_S_0.28_	4.1363	28.528
Sr_0.16_Bi_2_Se_2.6_S_0.4_	Sr_0.066_Bi_2_Se_2.63_S_0.36_	4.1342	28.517

## References

[b1] Kane C. L. & Mele E. J. Z_2_ topological order and the quantum spin Hall effect. Phys. Rev. Lett. 95, 146802 (2005). 16241681 10.1103/PhysRevLett.95.146802

[b2] Bernevig B. A., Hughes T. L. & Zhang S. C. Quantum spin Hall effect and topological phase transition in HgTe quantum wells. Science 314, 1757 (2006). 17170299 10.1126/science.1133734

[b3] Fu L. & Kane C. L. Superconducting proximity effect and Majorana fermions at the surface of a topological insulator. Phys. Rev. Lett. 100, 096407 (2008). 18352737 10.1103/PhysRevLett.100.096407

[b4] Zhang H. J. . Topological insulators in Bi_2_Se_3_, Bi_2_Te_3_ and Sb_2_Te_3_ with a single Dirac cone on the surface. Nat. Phys. 5, 438 (2009).

[b5] Qi X. L. & Zhang S. C. Topological insulators and superconductors. Rev. Mod. Phys. 83, 1057 (2011).

[b6] Wan X. G., Turner A. M., Vishwanath A. & Savrasov S. Y. Topological semimetal and Fermi-arc surface states in the electronic structure of pyrochlore iridates. Phys. Rev. B 83, 205101 (2011).

[b7] Wang Z. J., Weng H. M., Wu Q. S., Dai X. & Fang Z. Three-dimensional Dirac semimetal and quantum transport in Cd_3_As_2_. Phys. Rev. B 88, 125427 (2013).

[b8] Liu Z. K. . A stable three-dimensional topological Dirac semimetal Cd_3_As_2_. Nat. Mater. 13, 677 (2014). 24859642 10.1038/nmat3990

[b9] Huang S. M. . A Weyl Fermion semimetal with surface Fermi arcs in the transition metal monopnictide TaAs class. Nat. Commun. 6, 7373 (2015). 26067579 10.1038/ncomms8373 PMC4490374

[b10] Lv B. Q. . Observation of Weyl nodes in TaAs. Nat. Phys. 11, 724 (2015).

[b11] Inoue H. . Quasiparticle interference of the Fermi arcs and surface-bulk connectivity of a Weyl semimetal. Science 351, 1184 (2016). 26965625 10.1126/science.aad8766

[b12] Chong S. V., Williams G. V. M. & Moody R. L. The effect of manganese incorporation in Bi_2_Se_3_ on the thermal, electrical transport and magnetic properties. J. Alloy Compound. 686, 245 (2016).

[b13] Ruan J. . Ideal Weyl semimetals in the chalcopyrites CuTlSe_2_, AgTlTe_2_, AuTlTe_2_, and ZnPbAs_2_. Phys. Rev. Lett. 116, 226801 (2016). 27314733 10.1103/PhysRevLett.116.226801

[b14] Hor Y. S. . Superconductivity in Cu_*x*_Bi_2_Se_3_ and its implications for pairing in the undoped topological insulator. Phys. Rev. Lett. 104, 057001 (2010). 20366785 10.1103/PhysRevLett.104.057001

[b15] Zhang J. L. . Pressure-induced superconductivity in topological parent compound Bi_2_Te_3_. Proc. Natl. Acad. Sci. USA 108, 24 (2011). 21173267 10.1073/pnas.1014085108 PMC3017179

[b16] Wang M. X. . The coexistence of superconductivity and topological order in the Bi_2_Se_3_ thin films. Science 336, 52 (2012). 22422860 10.1126/science.1216466

[b17] Sun H. H. . Majorana zero mode detected with spin selective Andreev reflection in the vortex of a topological superconductor. Phys. Rev. Lett. 116, 257003 (2016). 27391745 10.1103/PhysRevLett.116.257003

[b18] Fu L. & Berg E. Odd-parity topological superconductors: Theory and application to Cu_*x*_Bi_2_Se_3_. Phys. Rev. Lett. 105, 097001 (2010). 20868184 10.1103/PhysRevLett.105.097001

[b19] Sasaki S. . Topological superconductivity in Cu_*x*_Bi_2_Se_3_. Phys. Rev. Lett. 107, 217001 (2011). 22181913 10.1103/PhysRevLett.107.217001

[b20] Lawson B. J., Hor Y. S. & Li L. Quantum oscillations in the topological superconductor candidate Cu_0.25_Bi_2_Se_3_. Phys. Rev. Lett. 109, 226406 (2012). 23368142 10.1103/PhysRevLett.109.226406

[b21] Levy N. . Experimental evidence for *s*-wave pairing symmetry in superconducting Cu_*x*_Bi_2_Se_3_ single crystals using a scanning tunneling microscope. Phys. Rev. Lett. 110, 117001 (2013). 25166563 10.1103/PhysRevLett.110.117001

[b22] Matano K., Kriener M., Segawa K., Ando Y. & Zheng G. Q. Spin-rotation symmetry breaking in the superconducting state of Cu_*x*_Bi_2_Se_3_. Nat. Phys. 12, 852 (2016).

[b23] Liu Z. H. . Superconductivity with topological surface state in Sr_*x*_Bi_2_Se_3_. J. Am. Chem. Soc. 137, 10512 (2015). 26262431 10.1021/jacs.5b06815

[b24] Han C. . Electronic structure of a superconducting topological insulator Sr-doped Bi_2_Se_3_. Appl. Phys. Lett. 107, 171602 (2015).

[b25] Shruti, Maurya V. K., Neha P., Srivastava P. & Patnaik S. Superconductivity by Sr intercalation in the layered topological insulator Bi_2_Se_3_. Phys. Rev. B 92, 020506(R) (2015).

[b26] Neupane M. . Electronic structure and relaxation dynamics in a superconducting topological material. Sci. Rep. 6, 22557 (2016). 26936229 10.1038/srep22557 PMC4776114

[b27] Pan Y. . Rotational symmetry breaking in the topological superconductor Sr_*x*_Bi_2_Se_3_ probed by upper-critical field experiments. Sci. Rep. 6, 28632 (2016). 27350295 10.1038/srep28632 PMC4923890

[b28] Du G. . Drive the Dirac electrons into cooper pairs in Sr_*x*_Bi_2_Se_3_. Nat. Commun. 8, 14466 (2017). 28198378 10.1038/ncomms14466 PMC5316857

[b29] Zhang C. J. & Oyanagi H. Local lattice instability and superconductivity in La_1.85_Sr_0.15_Cu_1−*x*_M_*x*_O_4_ (M = Mn, Ni, and Co). Phys. Rev. B 79, 064521 (2009).

[b30] Li L. . Coexistence of superconductivity and magnetism in K_*x*_Fe_2−*y*_Se_2−*z*_S_*z*_ (z = 0, 0.4). Phys. Rev. B 84, 174501 (2011).

[b31] Xiong J. . High-field Shubnikov-de Haas oscillations in the topological insulator Bi_2_Te_2_Se. Phys. Rev. B 86, 045314 (2012).

[b32] Bardarson J. H. & Moore J. E. Quantum interference and Aharonov-Bohm oscillations in topological insulators. Rep. Prog. Phys. 76, 056501 (2013). 23552181 10.1088/0034-4885/76/5/056501

[b33] Wang Y. . De Hass-van Alphen and magnetoresistance reveal predominantly single-band transport behavior in PdTe_2_. Sci. Rep. 6, 31554 (2016). 27516134 10.1038/srep31554 PMC4981858

[b34] Hu J. . Evidence of topological nodal-line fermions in ZrSiSe and ZrSiTe. Phys. Rev. Lett. 117, 016602 (2016). 27419579 10.1103/PhysRevLett.117.016602

[b35] Kushwaha S. K. . Sn-doped Bi_1.1_Sb_0.9_Te_2_S bulk crystal topological insulator with excellent properties. Nat. Commun. 7, 11456 (2016). 27118032 10.1038/ncomms11456 PMC4853473

